# Dietary and genetic effects on age-related loss of gene silencing reveal epigenetic plasticity of chromatin repression during aging

**DOI:** 10.18632/aging.100614

**Published:** 2013-11-14

**Authors:** Nan Jiang, Guyu Du, Ethan Tobias, Jason G. Wood, Rachel Whitaker, Nicola Neretti, Stephen L. Helfand

**Affiliations:** Department of Molecular Biology, Cell Biology and Biochemistry, Division of Biology and Medicine, Brown University, Providence, RI 02912, USA

**Keywords:** Aging, epigenetics, loss of gene silencing, constitutive heterochromatin, chromatin, calorie restriction, diet

## Abstract

During aging, changes in chromatin state that alter gene transcription have been postulated to result in expression of genes that are normally silenced, leading to deleterious age-related effects on cellular physiology. Despite the prevalence of this hypothesis, it is primarily in yeast that loss of gene silencing with age has been well documented. We use a novel position effect variegation (PEV) reporter in Drosophila melanogaster to show that age-related loss of repressive heterochromatin is associated with loss of gene silencing in metazoans and is affected by Sir2, as it is in yeast. The life span-extending intervention, calorie restriction (CR), delays the age-related loss of gene silencing, indicating that loss of gene silencing is a component of normal aging. Diet switch experiments show that such flies undergo a rapid change in their level of gene silencing, demonstrating the epigenetic plasticity of chromatin during aging and highlighting the potential role of diet and metabolism in chromatin maintenance, Thus, diet and related interventions may be of therapeutic importance for age-related diseases, such as cancer.

## INTRODUCTION

The phenotypic plasticity of aging highlights the importance of epigenetic factors such as chromatin remodeling in determining longevity. During aging, changes in chromatin state can alter gene transcription, resulting in expression of genes that are normally silenced, which has been postulated to result in deleterious effects on cellular physiology [1-6].

In eukaryotes, DNA associates with histones and protein complexes to form the compact structure of chromatin. The two major classes of chromatin, euchromatin and heterochromatin, differ in their degree of compaction and their accessibility for essential cellular functions such as gene transcription. Euchromatin is relatively decondensed, permitting transcription, while heterochromatin is more compact, leading to repression of transcriptional activity. Heterochromatin can be further categorized as constitutive or facultative. Constitutive heterochromatin occurs in the region of repetitive elements, including centromeres and telomeres, and is typically found in all cells throughout life. In *Drosophila*, the major constitutive heterochromatin regions are the pericentric regions of 3L, 2L, 2R, and the 4^th^ and Y chromosomes. Studies in yeast, nematodes and flies have shown changes in chromatin with age, and most excitingly, have demonstrated that molecular genetic and pharmacological interventions that are predicted to preserve or restore an earlier, more “youthful” chromatin state contribute to extend longevity [2, 3, 7-19].

While changes in chromatin during development have been extensively studied, it is only more recently that the dynamic nature of chromatin structure with age has begun to be investigated [1-3, 6, 17, 20-23]. In yeast and flies, it has been shown that specific heterochromatin regions are remodeled during aging, resulting in a loss of enrichment for repressive marks, especially in the mating type locus and subtelomeric regions in yeast [3], and in the pericentric regions and 4^th^ chromosome in flies [23]. During replicative senescence, human cells exhibit profound changes in chromatin remodeling, resulting in increased expression and mobilization of repetitive elements [6].

The decrease in repressive marks in yeast causes a loss of gene silencing near the telomere and in the mating type locus, and increased mobilization of the Ty1 transposable elements [3, 24]. These age-related changes in chromatin have deleterious consequences for the yeast cell. The decrease in repression in the mating type locus causes sterility in older yeast cells [2, 3, 7, 11] and increased mobilization of Ty1 elements leads to age-related genomic instability [24]. It has been postulated that similar changes in heterochromatin could have functional significance for gene expression in metazoans [23], especially loss of gene silencing with age.

As demonstrated by position effect variegation (PEV) in flies, the silencing of particular chromatin regions is in a dynamic state, especially during development. PEV results when a gene is located near a boundary between euchromatin (favoring gene expression) and heterochromatin (favoring gene repression), such that the same gene in different neighboring cells may stochastically find itself in a more euchromatic (expressed) state or more heterochromatic (repressed) state. In this sensitized boundary position, interventions that alter the level of active or repressive marks associated with euchromatin or heterochromatin can influence the degree to which the reporter gene is likely to be expressed. An increase in repressive marks is associated with repression of the PEV reporter gene, and conversely, a decrease in repressive marks is associated with increased expression of the PEV reporter gene among a population of cells [25, 26].

Although the relationship between age-related changes in repressive marks and loss of gene silencing has been demonstrated in yeast [2, 3, 7, 24], an age-related loss of gene silencing associated with loss of repressive heterochromatin marks has not been demonstrated in metazoans. Here we use two different PEV reporter lines associated with repressive heterochromatin that are capable of detecting loss of gene silencing at the level of single cells within specific tissues in individual adult flies, to examine whether there is a loss of silencing of gene expression with age in flies. We find a loss of gene silencing with age that is associated with an age-related decrease in repressive heterochromatin marks over the PEV reporter, which is opposed by dSir2 and CR. Diet “switch” experiments show a rapid effect of diet on gene silencing. These data suggest a close relationship between the life span-extending intervention CR and the maintenance of repressive heterochromatin during aging, underlining the importance of epigenetic plasticity in maintaining repressive heterochromatin, and in particular, the effect of diet and metabolism on chromatin states.

## RESULTS

### Gene silencing is lost with age in repressive heterochromatin regions of the adult fly

To test the hypothesis that loss of repressive heterochromatin marks with age leads to a loss of gene silencing in regions associated with repressive heterochromatin, we utilized a novel PEV reporter containing a heat shock-inducible *HS-lacZ* gene that allows for visualization of gene expression at the level of single cells, in individual tissues, at any time during the life of the adult fly [27, 28]. We examined two lines where the PEV transposon, containing a heat shock inducible *HS-lacZ* gene and mini-*white* gene, is located in association with one of two different heterochromatin regions. The *In(3L)BL1* line (BL1) is associated with 3L heterochromatin, while the *Tp(3;Y)BL2* (BL2) line transposon region has been translocated to the heterochromatic Y chromosome [27, 28]. Both lines demonstrate the PEV phenotype of mosaic eye pigmentation, caused by partial repression of the mini-*white* gene, and mosaic lacZ expression from PEV during developmental stages [27, 28]. As a control, we used the original *P[w+ HS-lacZ] (65E)* line from which the two PEV lines were derived, where the transposon is in the 3L euchromatin region [27, 28].

**Figure 1:**
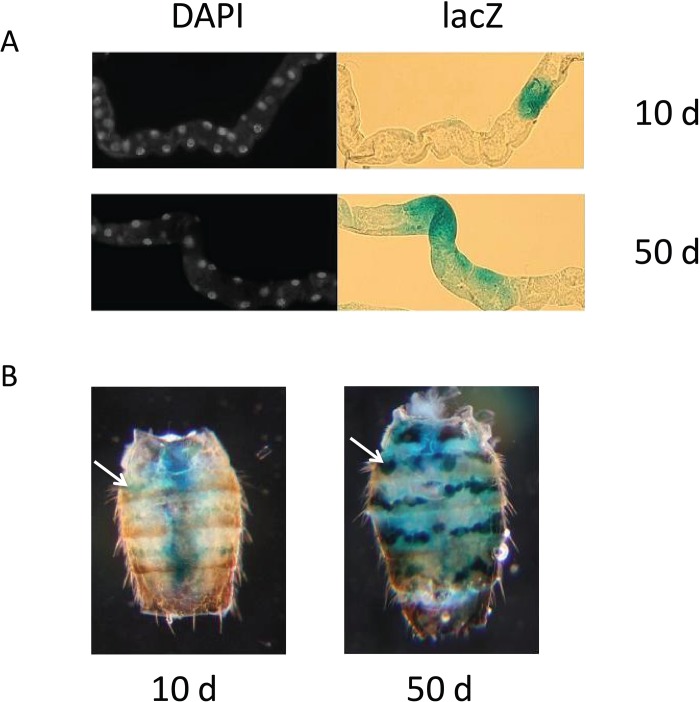
lacZ expression in PEV flies (**A**) Representative lacZ expression of Malpighian tubule cells from young, 10 day-old flies (upper panels) and old, 50 day-old flies (lower panels). Left panels show individual nuclei visualized with DAPI, shown in white; right panels show lacZ expression by X-gal staining of the same tubules, shown in blue. (**B**) Representative abdomens showing the blue lacZ expression of dorsal oenocytes in young, 10 day-old flies (left panel) and old, 50 day-old flies (right panel) from the BL2 line. The single white arrow in the left panel and the single white arrow in the right panel each point to an example of oenocytes positive for lacZ expression. Diffuse blue staining in central region of abdomen of 10 day-old fly in B is lacZ expression from bacteria in fly gut (Fig S1).

Upon a heat shock and recovery period (37°C for 1 hour, 25°C for 1 hour), flies of the control line, where the reporter construct is in normal euchromatin, were universally stained blue in all major tissues when reacted with X-gal to reveal lacZ expression, while the two PEV lines showed mosaic staining patterns, suggesting stochastic induction of HS-lacZ in individual cells by heat shock (Fig 1, S1).

We selected three distinctly different tissue types with ready accessibility to X-gal in whole mount preparations, and sufficient optical clarity to allow us to count individual stained and unstained cells in whole mount preparations. These included: Malpighian tubules (renal system), oenocytes (nutrient storage, pheromones) and the cells at the root of the major dorsal thoracic bristles (neuronal sensory and bristle support cells). Malpighian tubules, with their tubular, linearly aligned arrangement of cells, permit ready counting of individual lacZ-expressing cells as a percent of all cells present in the tubule, when these are visualized by a DAPI nuclear stain (Fig. 1, S1). Malpighian tubules have the additional distinction of being one of the few somatic tissues in the adult fly for which there are active stem cells and replacement of Malpighian tubule cells throughout adult life [29]. Oenocytes, with their linear and segmentally arranged distribution in the fly abdomen, can be easily counted with X-gal staining, as individually lacZ positive or negative, directly through the abdominal cuticle (Fig. 1, S1). The 10 major thorax bristles selected for counting (two pairs of dorsocentral setae, two pairs of postalar setae, and one pair of anterior scutellar setae) permit direct visualization of the cells at the root of their bristles, through the cuticle of the dorsal thorax.

**Figure 2:**
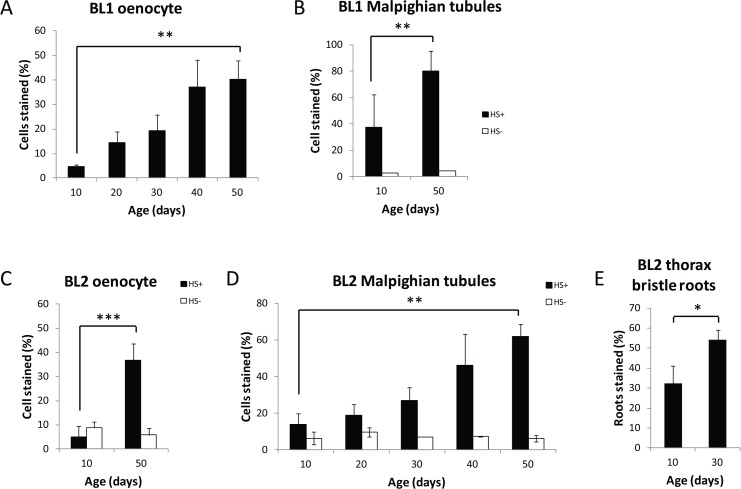
Age-related loss of gene silencing in heterochromatin regions Percent of HS-lacZ expressing cells increases with age in oenocytes (**A, C**) and Malpighian tubules (**B, D**) in BL1 and BL2 PEV lines, and in thoracic bristle roots in BL2 (**E**) (Black solid bars heat shocked=HS+). Three independent biological replicates from three separate cohorts were collected and examined for each age; a minumum of 15 flies per time point represent at least 75 flies (≥ 8,250 cells) total for oenocytes, and at least 30 flies (≥ 11,400 cells) total for Malpighian tubules. White open bars are the percent of HS-lacZ expressing cells without heat shock (HS-). BL1 Malpighian tubules were examined in only two separate biological replicates of 10 flies each. Without heat shock there is no lacZ expression in the BL1 oenocytes or in the thoracic bristle roots for BL1 and BL2 at age day 10 and day 50. Error bars represent SD, *P <0.05; **P < 0.01; and ***P<0.001. Neither BL1 nor BL2 show a non-heat shock, age-related increase of lacZ in flight or leg muscles as seen in euchromatin HS-lacZ reporters [40].

The level of cell-specific gene silencing with age was quantified by counting the number of lacZ-expressing cells as a percentage of total cells in each specific tissue. After heat shock, young 10-day old flies ranged from 5-35% of cells positive for lacZ expression in the Malpighian tubule, oenocytes, or thoracic bristle roots in the two different PEV lines, while similarly-treated 50-day old flies show between 40-80% lacZ expression in Malpighian tubule or oenocytes for BL1 and BL2 (Fig. 2). At present, the thoracic bristle cells can only be evaluated up to day 30, as visualization of lacZ expression in these cells is obscured by the age-associated increase in expression of the HS-lacZ reporter in the underlying thoracic muscles that is seen by day 30. Together, these data document the dynamic change in age-related loss of gene silencing in two different heterochromatin regions, 3L and Y heterochromatin.

### Basal level of activity of HS-lacZ is unaffected by age in Malpighian tubules and oenocytes

Our hypothesis is that the observed age-related loss of gene silencing is due to an age-related decrease in repressive heterochromatin in the region of the PEV reporters. Alternatively, an increase in the percent of cells expressing lacZ with age could result from a change in the basal level of activity of the HS promoter with age. We ruled out the possibility that the increase in percent of cells expressing lacZ with age seen in the PEV lines is the result of a change in the basal level of activity of the HS promoter with age by showing that without heat shock, lacZ expression in the BL1 and BL2 lines showed a low level of expression, ~10%, that did not change with age in these tissues (Fig. 2).

### Heat shock response of the HS-lacZ promoter is not age-sensitive

**Figure 3:**
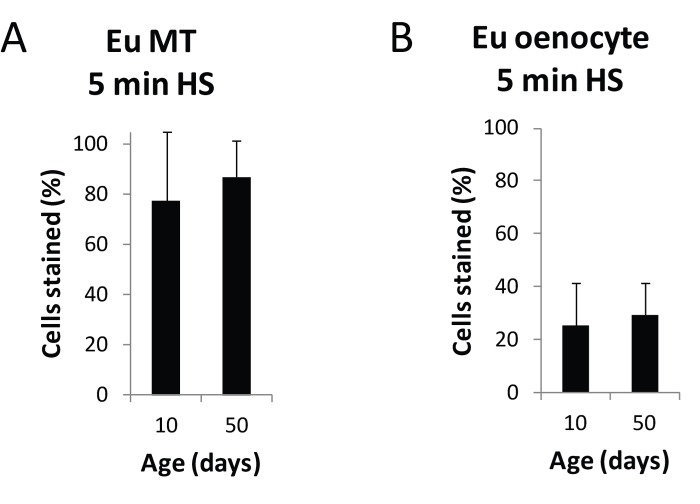
Heat shock response of the HS-lacZ promoter is not age-sensitive Following a 5 minute heat shock and X-gal staining of 10 day and 50 day old euchromatin HS-lacZ control line flies, total cells and lacZ expressing cells were counted in Malpighian tubules and oenocytes for three independent biological replicates from three separate cohorts. No significant difference in the percent of lacZ-positive cells in Malpighian tubules or oenocytes was seen with age. Each data point represents a minimum of 15 flies for a total of at least 30 flies (≥ 11,400 cells) for Malpighian tubules and at least 15 flies for a total of at least 30 flies (≥3,300 cells) total for oenocytes. Error bars represent SD.

To examine the possibility that the increase in lacZ expression with age could be caused by an age-related increased sensitivity of the HS promoter to heat shock, we examined the effect of age on the euchromatin control line, in which the reporter construct is free of the confounding effects of heterochromatin repression. We found no significant difference in lacZ staining with age in Malpighian tubules or oenocytes after a brief heat shock for the euchromatin control line (Fig. 3).

Taken together, these results suggest that the observed increase in lacZ expression with age in the BL1 and BL2 PEV lines is most likely due to an age-related de-repression of heterochromatin in the region of the reporter gene, and not to an increase in lacZ expression from an age-related increase in the basal activity of the *HS-lacZ* reporter, or to a direct effect of age on the sensitivity of heat shock-inducible promoter.

### Loss of gene silencing of lacZ and mini-*white* increases globally with age

**Figure 4:**
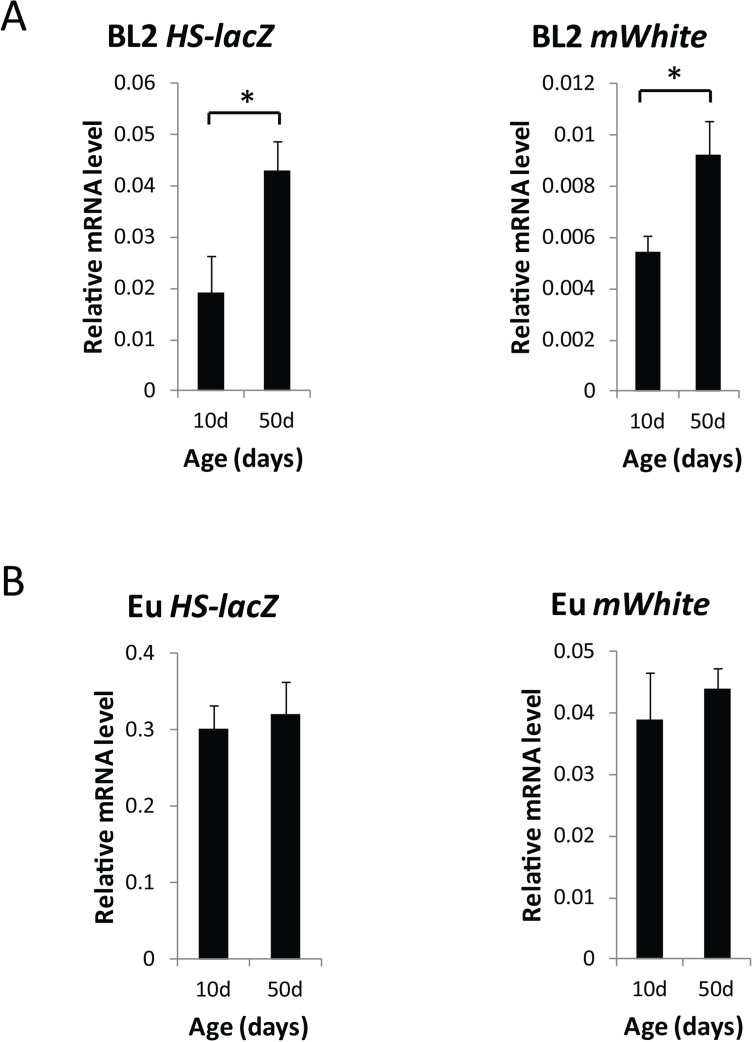
Global loss of gene silencing of the *HS-lacZ* gene and the *white* minigene in the heterochromatin-located PEV reporter construct, BL2 (**A**) *lacZ* mRNA levels in 10 day and 50 day old BL2 flies after heat shock and mini*-white* mRNA levels (*mWhite*) in 10 day and 50 day old BL2 flies without heat shock. (**B**) *lacZ* mRNA levels in 10 day and 50 day old euchromatin control flies after heat shock and mini*-white* mRNA levels (*mWhite*) in 10 day and 50 day old BL2 flies without heat shock. mRNA levels were normalized to *Gapdh1*. qPCR data are from three independent biological replicates, representing three separate cohorts of 30 flies each (≥90 flies total) used for qRT-PCR. Error bars represent SD, *P < 0.05. There was age-related change in the level of mRNA expression for the *HS-lacZ* [40] or *white* minigene when the reporter construct is located in euchromatin.

In addition to determining loss of gene silencing at the level of single cells in specific tissues of individual flies, we examined gene silencing with age globally, by measuring mRNA expression from the *HS-lacZ* gene and the mini-*white* gene that make up the PEV reporter construct [27, 28]. We found a two-fold increase in *HS-lacZ* (after heat shock) and mini-*white* (without heat shock) mRNA expression with age, demonstrating that for two different promoters, *HS-lacZ* and *white* minigene, both repressed by heterochromatin, there is a loss of silencing with age (Fig. 4). There is no change in level of mRNA expression for the *HS-lacZ* (after heat shock) or *white* minigene (without heat shock) when the reporter construct is in euchromatin (Fig 4). The finding of a two-fold increase in *HS-lacZ* and *white* minigene mRNA expression with age for the heterochromatin-positioned reporter further shows that in a variety of tissue types, there is a loss of gene silencing with age in heterochromatin regions.

### Loss of repressive heterochromatin marks is associated with loss of gene silencing with age

**Figure 5:**
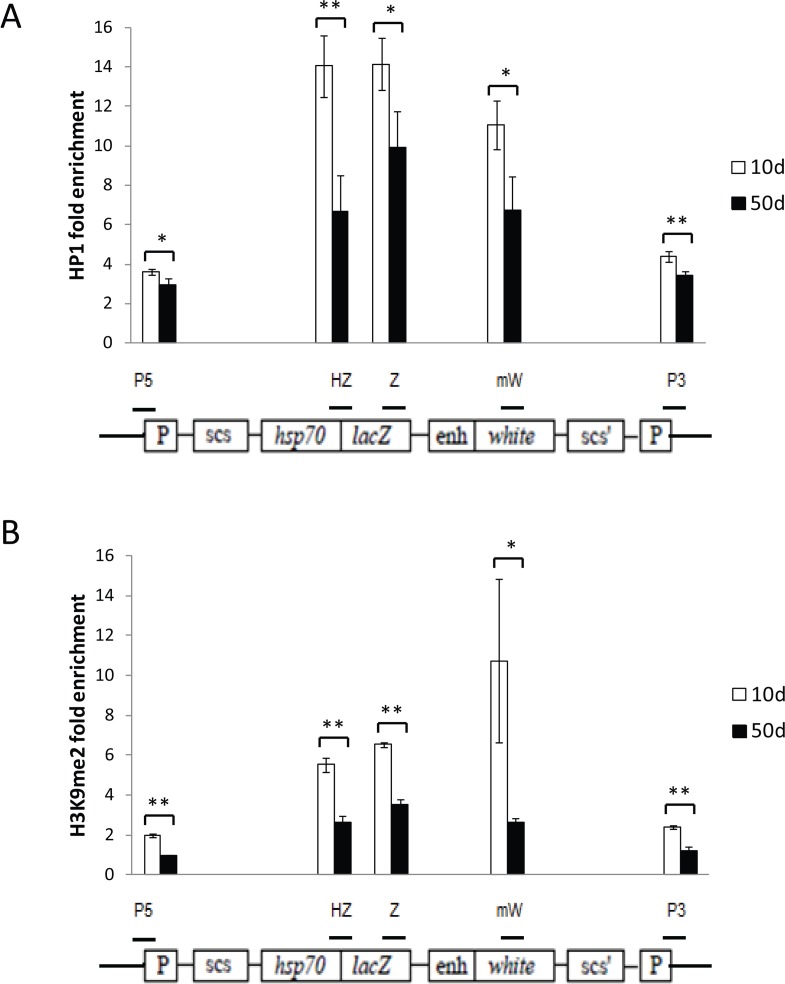
Age-related loss of gene silencing is associated with loss of repressive heterochromatin marks at the reporter construct Enrichment for HP1a (**A**) and H3K9me2 (**B**) in the reporter gene construct region, measured by ChIP-qPCR, decreases with age. Open bars represent 10-day old flies; solid bars, 50-day old flies. The location of the five regions of the reporter construct that were examined are depicted by the short horizontal bars over a diagram of the HS-lacZ reporter construct from [27, 28]: “P5” primers are specific for the 5' P-element LTR, “HZ” primers span the junction of the Hsp70 promoter and lacZ gene, “Z” primers are to an internal region of lacZ, “mW” primers are to an internal region of the mini-*white* gene, and “P3” primers are specific to the 3' LTR of the P-element construct. Enrichment values are the average for three independent biological replicates of ChIP-qPCR, normalized to the average of two different euchromatin control sites. Each data point represents three independent biological replicates of at least 100 flies from three separate cohorts. Error bars represent SD, *P < 0.05; **P < 0.01.

To determine whether the loss of gene silencing seen with age results from a decrease in heterochromatin repression in the region of the PEV construct, we measured relative enrichment of the repressive heterochromatin marks HP1a and H3K9me2 in the region of the PEV reporters, using chromatin immunoprecipitation and qPCR (ChIP-qPCR). We found a significant reduction in the enrichment of HP1a and H3K9me2 with age over the entire PEV construct (Fig. 5). Reduction in repressive heterochromatin marks over the PEV reporter was the same with or without heat shock, demonstrating that the heat shock used for inducing lacZ expression does not itself alter repressive heterochromatin marks in this region. Although it has been reported that heat shock leads to recruitment of HP1a to the native *hsp70* gene regions [30], we saw no further increased enrichment of HP1a in the *HS-lacZ* reporter region with heat shock. These data support the hypothesis that loss of gene silencing of the lacZ and white minigene reporters is likely due to a decrease in repressive heterochromatin with age.

### Increased expression of the histone deacetylase, *dSir2*, opposes age-related loss of gene silencing

**Figure 6:**
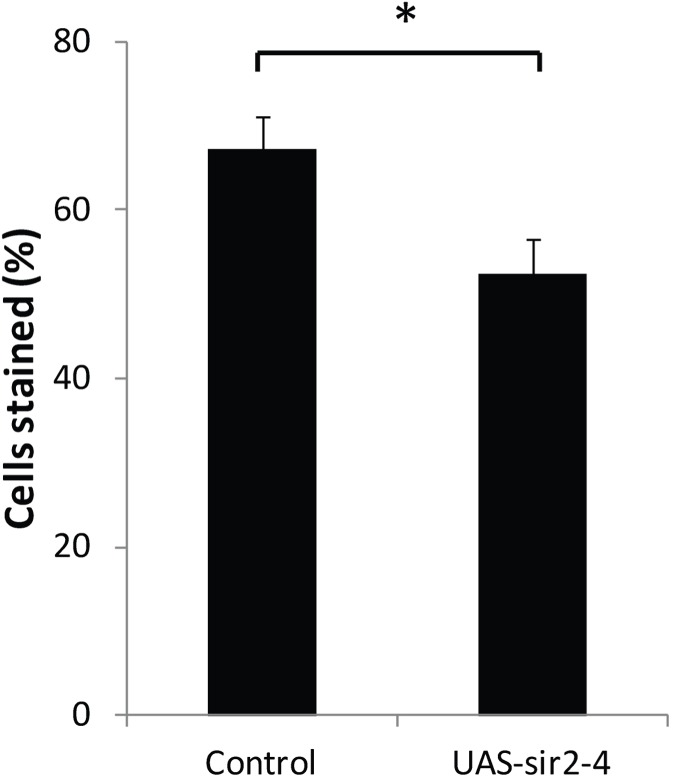
Increased dSir2 expression opposes age-related loss of gene silencing HS-lacZ BL2 reporter was crossed into *w^1118^* and *w^1118^/UAS-Sir2-4* flies (UAS-Sir2-4 backcrossed into *w^1118^* for 10 generations). The UAS-dSir2-4 flies have an extra copy of dSir2 that increases dSir2 expression without a Gal4 driver to ~150%, and extends life span [31]. At 20 days, HS-lacZ expression was quantified in oenocytes of *w^1118^* and *w^1118^/UAS-Sir2-4*. 3 independent staining experiments; error bars represent SD, *P < 0.05. Control is *w^1118^* background line crossed to BL2 HS-lacZ PEV reporter and UAS-sir2-4 is UAS-Sir2-4 line crossed to the BL2 HS-lacZ PEV reporter.

In yeast, the normal age-related reduction in expression of the histone deacetylase *Sir2* results in loss of gene silencing during aging that can be rescued by increasing *SIR2* expression [3]. To examine the effect of increased *dSir2* expression on the age-related loss of gene silencing in *Drosophila*, we measured and compared loss of gene silencing with age in our PEV reporter line containing the normal two copies of dSir2, and in flies of the same PEV line, but with an added copy of a dSir2 transgene [31], which increased *dSir2* expression (measured by qPCR) level to approximately 150% of control. Elevated *dSir2* expression led to a statistically significant decrease in lacZ-expressing cells by day 20 in oenocytes (Fig. 6). Thus, increasing the level of expression of a known histone deacetylase enzyme, *dSir2*, may delay age-related loss of gene silencing in flies as it does in yeast [3].

### Calorie restriction delays the age-related loss of gene silencing

**Figure 7:**
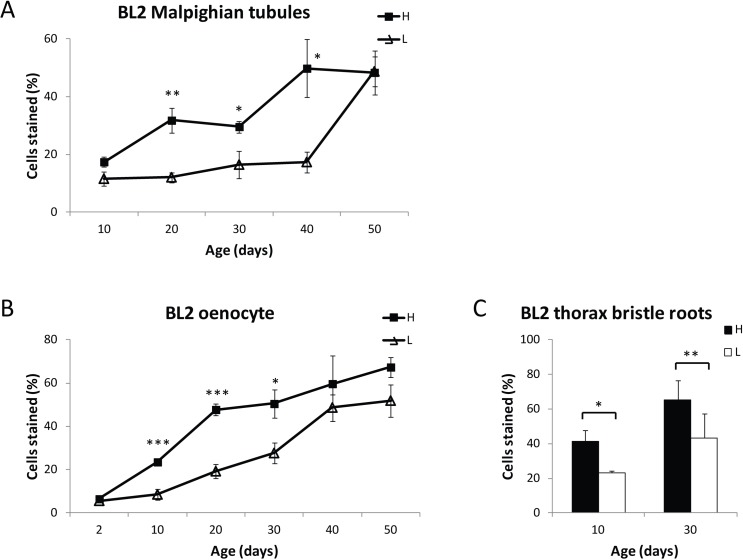
Dietary restriction delays age-related loss of gene silencing BL2 flies fed on a high (H) or low (L) calorie diet were stained at various ages, and HS-LacZ expression was quantified for Malpighian tubules (**A**) oenocytes (**B**) and thoracic bristle roots (**C**). Each data point in (**A**) and (**B**) represents 5 biologically independent experiments from different cohorts (25 or more flies for each time point; at least 300 flies (≥33,000 cells) total were scored for oenocytes, and at least 250 flies (≥ 95,000 cells) total were scored for Malpighian tubules); error bars represent SEM, *P < 0.05; **P < 0.01, ***P < 0.001. For thoracic bristle roots, BL2 flies fed on a high (H) or low (L) calorie diet were heat shocked, stained at Day 10 and Day 30 with X-gal, and HS-LacZ expression was quantified for the cells at the base of 10 thoracic bristles. Three independent biological replicates of 15 flies for each time point representing 60 flies (> 600 bristle roots) total were counted. Error bars represent SD, *P < 0.05; **P < 0.01.

To further investigate the relationship between aging and loss of gene silencing, we examined the effect of the life span-extending intervention CR on age-associated loss of gene silencing by comparing the loss of gene silencing with age in Malpighian tubules, oenocytes, and thorax bristle root cells for flies grown on high or on low calorie food (Fig. 7). At an early age, flies on either high or low calorie food showed the same percent of stained cells (Fig. 7). CR resulted in significantly lower levels of age-related *HS-lacZ* loss of gene silencing from ages 20 through 40 days for Malpighian tubules and ages 10 through 30 days for oenocytes, after which time, the maximal level of lacZ staining for the PEV line in each of these tissues is reached. The age of increase in loss of gene silencing is delayed under low calorie conditions, as indicated by the finding of a statistically significant increase in loss of gene silencing in oenocytes on high calorie food from day 2 to day 10 (P<0.001) and from day 10 to day 20 (P<0.001), as well as a statistically significant increase in loss of gene silencing in Malpighian tubules on high calorie food from day 10 to day 20 (P<0.05) that is not seen until a later time on low calorie food (Fig. 7). These analyses indicate that CR delays the age-related loss of gene silencing, providing strong evidence for the hypothesis that decreases in repressive heterochromatin and loss of gene silencing are a component of the aging process.

### Diet switch experiments demonstrate the plasticity of the adult chromatin state

The finding that CR delays age-related loss of gene silencing suggests a relationship between CR life span extension and maintenance of repressive heterochromatin. However, it does not distinguish between CR having a direct effect on maintenance of repressive heterochromatin, or an indirect effect, by slowing down the rate of aging. To test whether CR may directly mediate repressive heterochromatin maintenance, we measured the level of gene silencing in flies switched from high calorie food to low calorie food and vice versa (diet switch experiments), changes known to rapidly change mortality rates [32],. We found that within 72 hours after either 20 or 30 day-old *HS-lacZ* PEV reporter flies (BL2) are switched from a low to a high calorie food, or from a high to low calorie food, the percent of cells showing lacZ expression in tissues of these “switched” flies changes toward the level of gene silencing for flies that have been kept on the “switched-to” diet throughout their lives.

**Figure 8:**
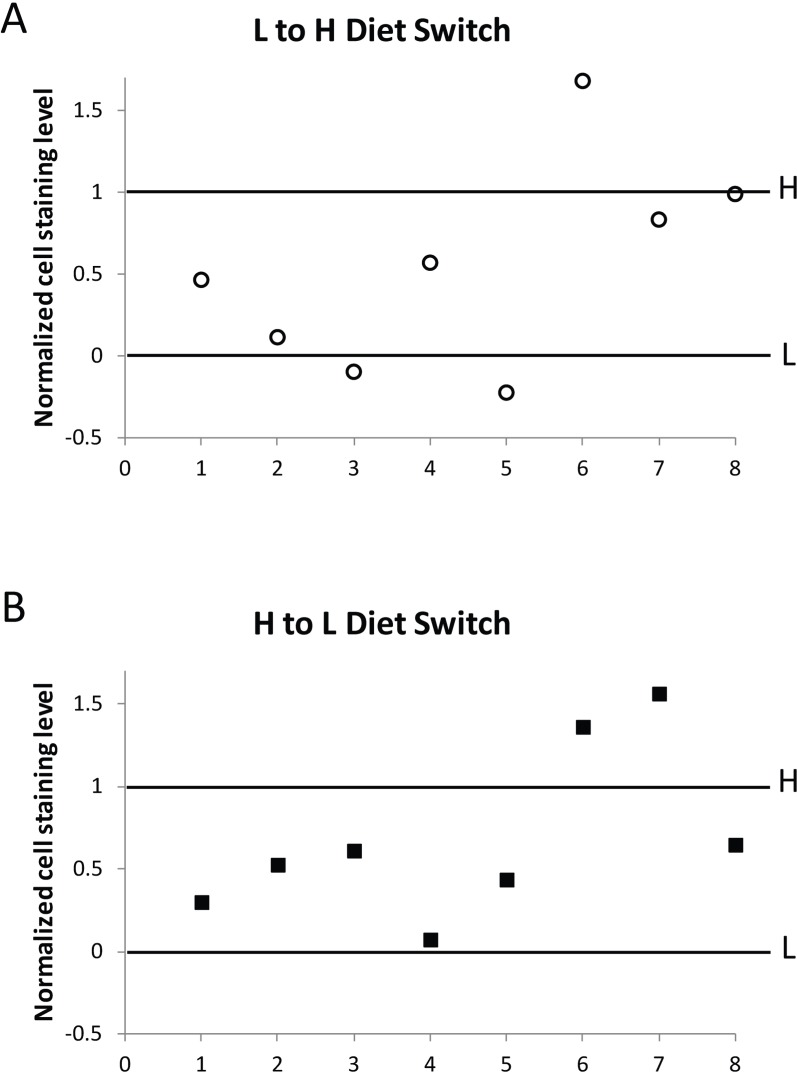
Change in HS-lacZ silencing in response to diet switch In eight independent diet switch experiments (shown individually as 1-8 in the X-axis of **A** and **B**), BL2 flies at day 20 to 30 that had been fed on low calorie food were transferred to high calorie food (L to H, A), and separately, flies at the same age that had been fed on high calorie food were transferred to low calorie food (H to L, B). After 72 hours on the new food, five flies from each group in each individual experiment (1-8) were assessed for HS-lacZ silencing, together with, and normalized to, flies grown solely on high calorie (H) and low calorie (L) food without diet switch. In normalization, oenocyte staining level for flies continuously kept on low calorie food, “L”, was set as 0, and for flies continuously kept on high calorie food, “H”, as 1, for each individual switch experiment. For each biological replicate, L to H and H to L values are represented as percent of the total difference between values for flies living continuously on high calorie food and on low calorie food. Primary data in Table S1 and statistical analyses in Table S2.

To illustrate the change following diet switch for each of the eight biological replicates, we plotted the percent of lacZ expression in oenocytes for flies switched from low calorie to high calorie food, or from high calorie to low calorie food, as a percent of the total difference between the lacZ expression value for flies living continuously on high calorie food (1.0), and the value for flies living continuously on low calorie food (0), for each of the biological replicates (Fig. 8). We performed two-sided chi square analyses on the individual replicates as well as on the pooled data, using the raw counts of the number of positive cells and negative cells within the context of a binomial comparative trial model to determine whether the change in the level of gene silencing for the switched flies is in the same direction as the level of gene silencing for flies fed continuously on the diet to which they were switched. Analysis of the pooled data showed a statistically highly significant change away from the level of gene silencing of the food they had been living on (P<0.005) and toward the level of gene silencing they were moved to. Analysis of the individual replicates showed that for those flies moved from high calorie to low calorie food, 6 of the 8 replicates exhibit a statistically significant change in gene expression away from the level of gene expression of flies on high calorie food (P≤0.02) while 6 of the 8 replicates moved from low calorie to high calorie food exhibited a statistically significant change in gene expression away from the level of gene expression of flies on low calorie food (P≤0.05). The finding that flies at 20 or 30 days of age can rapidly (within 72 hours) respond to a change in diet by altering the level of gene silencing of a PEV reporter gene, a direct functional measure of the state of repressive heterochromatin, suggests a close relationship between the life span-extending intervention CR, and maintenance of repressive heterochromatin. Furthermore, the rapidity of the response to diet seen in the change in gene silencing demonstrates the epigenetic plasticity of chromatin during aging and the potential role of diet and metabolism in chromatin maintenance.

## DISCUSSION

The loss of repressive heterochromatin with age, a concomitant decline in gene silencing, and the emergence of deleterious gene expression, including expression and mobilization of Ty1 transposable elements, have been demonstrated in replicative aging in yeast [3, 24]. The finding of age-associated remodeling of chromatin in repressive constitutive heterochromatin regions in flies and mammals suggests that a loss of gene silencing with age in these regions could also play a role in metazoan aging [6, 23]. In flies, age-related chromatin remodeling has been shown to include a decrease in enrichment of two of the primary repressive heterochromatin marks, HP1a and H3K9me3, in the constitutive heterochromatin regions [23], but has not been shown to result in loss of gene silencing in these regions. We used a novel PEV reporter gene in flies that allows for direct visualization of loss of gene silencing in individual cells within different tissues and organs, in adult flies with age. Two different lines, with the PEV reporter located in the 3L or Y chromosome heterochromatin regions, demonstrated that a decrease in enrichment for repressive marks in heterochromatin [23] affects gene silencing in these regions. We found that a consequence of a decline in repressive heterochromatin regions with age is a progressive, graded loss of gene silencing in constitutive heterochromatin regions. In metazoans such an age-related decrease in repressive heterochromatin could contribute to cellular aging as a result of the inappropriate expression from normally silenced genes, non-coding transcriptional units and transposable elements residing in these regions.

In yeast, the age-related loss of gene silencing in the mating type locus and telomeric regions is associated with a decline in the expression of the *SIR2* gene. Conversely, when Sir2 levels are increased, it delays the age-related loss of repressive marks and the loss of gene silencing that leads to harmful gene expression (e.g. mating type locus expression and sterility) [3, 18, 20]. Using the *Drosophila* age-associated PEV reporter and an added *dSir2* transgene, we were able to show that, as in yeast, the level of expression of *dSir2* in flies affects loss of gene silencing in heterochromatin. *dSir2* expression at approximately 150% of control levels, sufficient to extend life span [31], opposes the age-related loss of gene silencing of the PEV reporter. These data provide evidence that *dSir2* plays a role in maintaining repressive heterochromatin, and that it can delay the loss of gene silencing with age in metazoans, as it is known to do in yeast.

CR is an intervention that has been demonstrated to extend life span in many different species, including yeast, nematodes, flies and mammals, and to delay the onset of most age-related pathologies in mammals. CR is often used as a test of the functional relevance of an observed age-associated change to the aging process. Failure of CR to delay an age-associated phenotype is evidence against the potential relevance of that phenotype to functional aging. Using the PEV reporter in the context of high and low calorie diets, we examined three different fly tissues: oenocytes, bristle root cells, and Malpighian tubules. Oenocytes and bristle root cells are made up entirely of post-mitotic cells, while Malpighian tubules as one of the few organs in the fly with stem cells, have cells that are constantly being replaced [29]. In all three tissues examined, CR significantly delayed loss of gene silencing with age, indicating that loss of gene silencing in the heterochromatin region may be a physiological change that is functionally relevant to the process of aging.

To further examine the linkage between CR and loss of gene silencing with age, a dietary switch experiment was performed where gene expression in individual oenocytes was assessed with age in the PEV reporter flies. We found that flies moved from a high to a low calorie diet undergo a rapid switch toward the more repressed, youthful condition of flies living on a low calorie diet throughout life. Conversely, flies moved from a low to a high calorie diet show an increase in the number of cells expressing the PEV reporter gene, suggesting a less repressed, more open chromatin state in the region of the reporter, toward the direction of flies living on a high calorie diet throughout life. The speed at which the gene expression changes occur, within 72 hours, both for flies moved from a high to a low calorie diet, and for flies moved from a low to a high calorie diet, implies a close relationship between CR and the mechanisms responsible for determining the state of repressive heterochromatin. These results serve to demonstrate the epigenetic plasticity of the chromatin state during aging, and the importance of understanding the role of dietary and other physiological alterations on chromatin maintenance and cellular aging.

Our studies on age-related loss of gene silencing in oenocytes provide insights into the role of cell autonomous and non-cell autonomous factors in determining the state of chromatin and the regulation of gene expression during aging. In cell autonomous aging, the accumulation of damaged or altered macromolecules within the cell over time is thought to lead to cellular dysfunction, senescence and aging [33-36]. As such, the cell autonomous theory is often associated with long-lived cells that remain and function within the organism throughout most of adult life, permitting time to accumulate damage. Examples of long-lived cells include neurons and cardiac cells in mammals, most of the cells in adult flies and nematodes, and stem cells. Alternatively, in non-cell autonomous aging, systemic or local humoral factors from outside the cell itself play a prominent role in influencing cellular aging. These distinctions have practical relevance in the search for therapeutic interventions aimed at restoring the vibrancy of older tissues or organs. If cellular aging were due solely to progressive, irreversible cell autonomous events, then rejuvenation of a tissue or organ might be most successful through the introduction of new, young cells into that tissue or organ, as advocated by stem cell therapy. However, if in some tissues and organs, components of cellular aging are non-cell autonomous factors, then the administration of exogenous humoral factors might be utilized to reverse the aged conditions of these cells and organs, thus restoring them to a more youthful and healthy state of functioning.

Adult oenocytes, which are one of the major sites for intermediary metabolism, nutrient storage and pheromone production in the fly [37], comprise a tissue made up of post-mitotic cells that have resided in the adult since the emergence of the fly into its adult state, and that lacks addition or replenishment by new cells during adult life. As an example of cellular aging of a tissue consisting of long-lived post-mitotic cells, oenocytes might be anticipated to demonstrate cell autonomous aging. However, the diet switch studies, in which a simple change in diet rapidly alters the state of chromatin repression and gene expression in these cells, indicates that non-cell autonomous factors can also influence the state of heterochromatin repression in long-lived, fully differentiated post-mitotic cells. The relatively modest dietary intervention we utilized affects chromatin and loss of gene silencing in the same direction and with the same rapidity that it affects mortality, for example, decelerating mortality and restoring loss of gene silencing toward a more youthful state when flies are moved to a lower calorie diet. These studies serve to demonstrate the epigenetic plasticity of chromatin maintenance and its functional significance to age-related loss of gene silencing. Furthermore, the rapid alteration in chromatin states in response to moderate changes in diet highlights the utility of diet and other related therapeutic interventions as a method of restoring beneficial chromatin states in order to delay the development or progression of age-related disorders, including cancer.

## METHODS

### Fly lines and medium

The two PEV lines, *In(3L)BL1* (BL1) and *Tp(3;Y)BL2* (BL2), and the euchromatin control line *P[w+ HS-lacZ] (65E)* (Eu) were a gift from Joel C. Eissenberg and are described in [27, 28]. The Sir2 *UAS-Sir2-4* transgenic line was backcrossed to a *w^1118^* control line for 10 generations [31]. Flies were grown and kept at 25 °C on standard cornmeal food (approximately 12% sucrose, 3% yeast, 5% corn meal, and 1% agar). For studies on dietary restriction, after eclosion, flies were grown on either high calorie 1.5N (1.5N: 15% sucrose, 15% yeast, 5% corn meal, 2% agar), or low calorie (0.5N: 5% sucrose, 5% yeast, 5% corn, 2% agar) food, similar to [38].

### Quantification of HS-lacZ expression

Flies were collected and heat shocked [1h at 37 °C, followed by 1h recovery at 25 °C for Malpighian tubules, 40 min heat shock at 37 °C and 40 min recovery for oenocytes, and 10 min heat shock at 37 °C and 30 min recovery for thorax bristle roots], fixed with 4% paraformaldehyde in PBS for 10 min, washed in PBS, and incubated in X-gal staining solution [39] at room temperature overnight. Presence of blue staining of a cell represented a positive lacZ-expressing cell. Different levels of intensity of blue staining were not determined, only presence or absence of blue staining was noted.

Percent of stained Malpighian tubule cells was determined as fraction of stained cells out of total nuclei, visualized by DAPI. Because of the possibility that new cells, migrating from stem cells in the proximal region of the Malpighian tubule [29], could confound the age-related changes in loss of gene silencing, proximal - distal position of lacZ positive and negative cells in the Malpighian tubules was determined. We found no evidence of a positional bias for either lacZ expression or its absence with age, minimizing the possibility that clonality in lacZ expression or distribution related to proximal or distal origin of the cells skewed our results.

The percent of stained oenocytes was determined from days 20-50 by counting lacZ-stained and total brown-pigmented oenocytes in the six dorsal-abdominal segments. Brown-pigmentation is seen by day 20 in BL1, BL2 and control flies. The total number of oenocytes days 2-10 was shown to be the same as days 20-50 by counting the number of lacZ-expressing oenocytes in the control line after heat shock days 2-50 (no significant difference was seen with age) and comparing with the number of brown-pigmented oenocytes in unstained cohorts of control, BL1 and BL2 flies at days 20-50 (no significant differences with age or line). Therefore, the percent lacZ staining for the Day 2-10 was determined by counting the number of lacZ-stained oenocytes in the six dorsal-abdominal segments and comparing them to a “standard” number of oenocytes in each segment: 1=19, 2=22, 3=24, 4=24, 5=20, and 6=18 cells +/− 1.5 cells/segment.

The percent of stained thoracic bristle roots was determined by counting the number of stained thoracic bristle roots of 10 examined (two pairs of dorsocentral setae, two pairs of postalar setae, and one pair of anterior scutellar setae), by direct visualization of the cells at the root of their bristles, through the cuticle of the dorsal thorax.

### Chromatin immunoprecipitation (ChIP) and qPCR

For each chromatin sample, 100 male flies were homogenized in PBS, and cross-linked in 1.8% formaldehyde at RT for 10 min. Cross-linking was stopped by addition of glycine (final concentration 0.25M) for 5 min. Subsequent washing, sonication and chromatin immunoprecipitation followed the protocol described in [23]. Anti-HP1a antibody C1A9 was obtained from the Developmental Studies Hybridoma Bank; anti-H3K9me2 antibody ab1220 was purchased from Abcam. qPCR primers were designed for five unique sites spanning the construct length, as well as for the two control sites in euchromatin regions (2L: 22341800 and 3L: 13168600), which were shown to be free of heterochromatin marks in both young and old flies [23].

Primers: P5-F: TACTTCGGTAAGCTTCGGCTATCG; P5-R: CGTGTCACACTAGAATCTTCA; HZ-F: GAAGAACTCACACACAATGCC; HZ-R: AAGGGGATTAAGTTGGGTAACGCC; Z-F: TGCGGGACGCGCGAATTGAATTAT; Z-R: AGATGGCGATGGCTGGTTTCCAT; mW-F: GGTAGCTGTGCTCGCTATATAAGACA; mW-R: ACGAAGTATCCTACGAAGTAGGTTTA; P3-F: ACGTTAAGTGGATGTCTCTTGCCG; P3-R: GCAGGTTGCCCAAGAAGATTCGAC; CTRL1-F: GCCCTGGTGGAAGTGGAAATTGAA; CTRL1-R: GGCTCAGAAACAGAAGCAGGAACA; CTRL2-F: TAGAAAGATCTGAGGAGAGTCCGCCT; CTRL2-R: TAACATGGCCCTCGGCTACGATTT. CTRL1 is at chromosome position: 3L: 22341700 and CTRL2 is at chromosome position: 2L: 13168600. Enrichment for target sites and euchromatic control sites in ChIP DNA relative to input was determined by qPCR (SYBR Green PCR Master Mix, Applied Biosystems). The fold enrichment was derived from ΔΔCt between each target site and the average of the two control sites.

### RNA isolation and RT-PCR

mRNA was isolated from whole flies using Dynabeads mRNA DIRECT Kit (Invitrogen). cDNA was synthesized using iScript cDNA Synthesis Kit (Bio-Rad). *HS-lacZ* and *mini-white* mRNA levels were normalized to *gapdh1*. For the heat shock condition, flies were heat shocked at 37 °C for 30 minutes.

Primers: HS-lacZ-F: TGCGGGACGCGCGAATTGAA TTAT; HS-lacZ-R: AGATGGCGATGGCTGGTTTCC AT; mWhite-F: TTTGGGCCAACAACTCACGCAA; mWhite-R: CTCAGGTGAACACATTTATCGTGGC; Gapdh1-F: TTAACCTGGACGCCTACAGCC; Gapdh1-R: CCTCGACGATCTCGAAGTTGTCAT.

## SUPPLEMENTARY DATA


