# Supplementary figures and images for: Dietary and genetic effects on age-related loss of gene silencing reveal epigenetic plasticity of chromatin repression during aging
Source: Aging (Albany NY). 2013 Nov 14;5(11):813–24. doi: 10.18632/aging.100614 (PMC3868724; doi:10.18632/aging.100614)

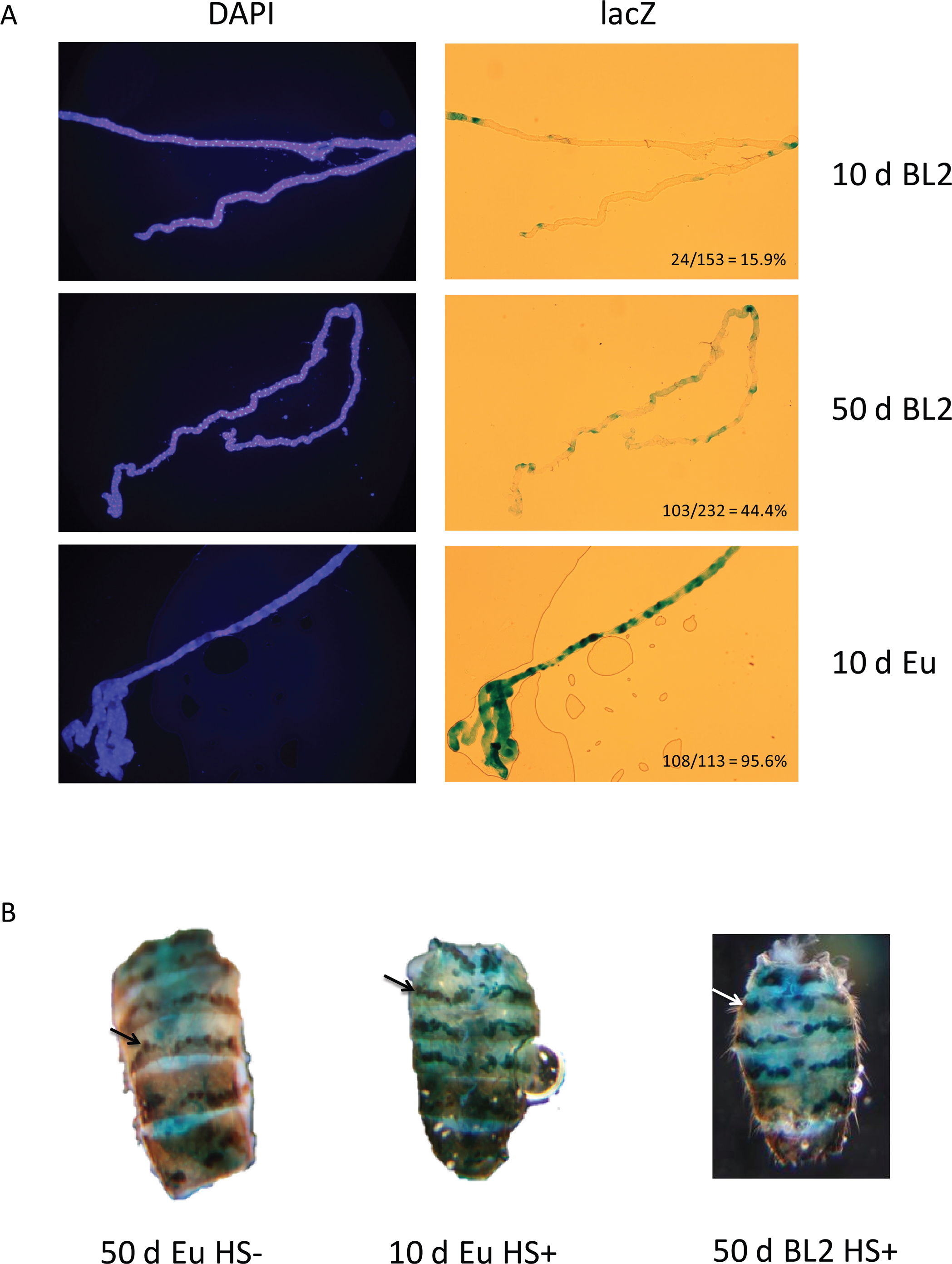

Supplement: Supplementary file 1 [file aging-05-813-s001.tif]
